# The bounds estimate of sub-band operators for multi-band wavelets

**DOI:** 10.1186/s13660-018-1630-1

**Published:** 2018-02-17

**Authors:** Qingyun Zou, Guoqiu Wang, Qian Cao

**Affiliations:** 1 Hunan Province Cooperative Innovation Center for The Construction Development of Dongting Lake Ecological Economic Zone, Hunan University of Arts and Science, Changde, P.R. China; 2 College of Mathematics and Computer Science, Hunan Normal University, Changsha, P.R. China

**Keywords:** 42C40, 42A16, Multi-band wavelets, Circular matrix, Biorthogonality, Sub-band operators, Bounds estimate

## Abstract

A concept of the sub-band operator of multi-band wavelets is introduced, the theory of d-circular matrices is developed and the upper bound and the lower bound of the norm of the sub-band operator are obtained. Examples are provided to illustrate the results proposed in this paper.

## Introduction

During the last three decades, the theory of frames, which generalize the notion of bases by allowing redundancy yet still providing a reconstruction formula, has been growing rapidly, since several new applications such as nonlinear sparse approximation (e.g., image compression), coarse quantization, data transmission with erasures, and wireless communication, have been developed [1–7]. As a special class of frames, the multi-band wavelets have attracted considerable attention due to their richer parameter space, to give better energy compaction than 2-band wavelets [8–16].

Let *H* be a separable Hilbert space, and *E* an indexing set. A sequence $\{f_{l}\}_{l\in E}$ is called a frame in *H* if there exist constants $0< A\leq B<+\infty$ such that

1.1 $$ A\|f\|^{2}\leq\sum_{l\in E}\big|\langle f,f_{l}\rangle\big|^{2}\leq B\|f\|^{2}, \quad\forall f\in H, $$

where *A* and *B* are called lower and upper frame bounds, respectively. If $A=B$, the frame is called tight frame.

When $H=L^{2}(R)$, a wavelet $\psi(x)\in H$ gives rise to a classical wavelet frame $\{a^{-j/2}\psi(a^{-j}x-bk),j,k\in Z\}$ with parameters $a>1$, $b>0$. Chui and Shi [17] established the relationship between the parameters and the frame bounds,

1.2 $$ A\leq\frac{1}{2b\ln a} \int_{-\infty} ^{+\infty}\frac {|\widehat{\psi}(\omega)|^{2}}{|\omega|}\,d\omega\leq B. $$

Equation (1.2) shows that the energy of a biorthogonal wavelet transform is controllable although it is not conservative. Moreover, $B-A$ or $\widetilde{B}-\widetilde{A}$ are smaller, the performance of a biorthogonal wavelet transform may be better, i.e., the energy is amplified in some cases and decreased in other cases. The classical biorthogonal wavelets are not tight frames, so obtaining the exact values of their bounds is difficult. Instead of estimating the bounds in (1.2), we can try to obtain the upper bound and the lower bound of the norm of the sub-band operator.

This paper is organized as follows. In Sect. 2, we define the sub-band operator, and obtain the limit form of the norm of the sub-band operator. We present a method for computing the upper bound and the lower bound of the norm of the sub-band operator based on the theory of circular matrix. Section 3 gives some examples to illustrate the results proposed in this paper.

## Sub-band operator and *d*-circular matrix

Recall the sub-band coding scheme or Mallat algorithm associated to a *d*-band real biorthogonal wavelets. There are 2*d* filters $h=(h_{n})_{n\in Z}$, $g^{i}=(g^{i}_{n})_{n\in Z}$ ($i=1,2,\ldots,d-1$), $\widetilde{h}=(\widetilde{h}_{n})_{n\in Z}$ and $\widetilde{g}^{i}=(\widetilde{g}^{i}_{n})_{n\in Z}$ ($i=1,2,\ldots,d-1$), $\{h,g^{1},g^{2},\ldots,g^{d-1}\}$ are used for decomposition and $\{\widetilde{h},\widetilde{g}^{1},\widetilde{g}^{2},\ldots,\widetilde {g}^{d-1}\}$ for reconstruction. Starting from a data sequence $x=(x_{n})_{n\in Z}$, we convolve with $\{h,g^{1},g^{2},\ldots,g^{d-1}\}$,

2.1 $$ \begin{gathered} c_{n}=\sum_{k}h_{dn-k}x_{k}, \\ p^{i}_{n}=\sum_{k}g^{i}_{dn-k}x_{k},\quad i=1,2,\ldots,d-1. \end{gathered} $$

The reconstruction operation is

2.2 $$ \widetilde{x}_{k}=\sum_{n}\Biggl( \widetilde{h}_{dn-k}c_{n} + \sum^{d-1}_{i=1} \widetilde{g}^{i}_{dn-k}p^{i}_{n}\Biggr). $$

The constraint conditions for biorthogonal *d*-band filter banks with perfect reconstruction property are:

- the low-pass and high-pass condition
  $$\sum h_{k}=\sum\widetilde{h}_{k}= \sqrt{d},\qquad \sum g^{i}_{k}=\sum \widetilde{g}^{i}_{k}=0,\quad i=1,2,\ldots,d-1; $$
- the biorthogonal condition
  $$\sum_{k} h_{k}\widetilde{h}_{k+dj}= \delta_{j},\qquad h_{k}\widetilde {g}^{i}_{k+dj}=g^{i}_{k} \widetilde{h}_{k+dj}=0,\quad\quad g^{i}_{k}\widetilde {g}^{l}_{k+dj}=\delta_{i-l}\delta_{j}, $$
  where $\delta_{j}$ denotes the *Dirac* sequence such that $\delta_{j}=1 $ for $j=0$ otherwise $\delta_{j}=0 $;
- the perfect reconstruction condition $x=\widetilde{x}$.

In order to define (2.1) and (2.2) as operators, namely, sub-band operators, we assume that the input signal $x\in l^{2}(-\infty,+\infty)$. Now consider the separable Hilbert space $l^{2}(-\infty,+\infty)$. Define

$$(x,y)=\sum_{n=-\infty}^{+\infty}x_{i} \overline{y}_{i} $$

as the usual inner product on $l^{2}(-\infty,+\infty)$, where $x,y\in l^{2}(-\infty,+\infty)$, and $\overline{y}_{i}$ denotes the conjugate of the complex number $y_{i}$.

### Definition 2.1

The operator

$$T:Tx=y $$

is called a sub-band operator, where $x\in l^{2}(-\infty,+\infty)$, $y=(\ldots,c_{0},p^{1}_{0},p^{2}_{0},\ldots ,p^{d-1}_{0},c_{1},p^{1}_{1}, p^{2}_{1},\ldots,p^{d-1}_{1},\ldots)$, $c_{n}$ and $p^{i}_{n}$, $i=1,2,\ldots,d-1$ are defined as in (2.1).

Throughout this paper, we assume $h,g^{1},g^{2},\ldots,g^{d-1},\widetilde{h},\widetilde{g}^{1},\widetilde {g}^{2},\ldots,\widetilde{g}^{d-1}$ have only finitely many nonzero elements.

### Theorem 2.1

*If the* *d*-*band biorthogonal wavelets determined by the filters* $h,g^{1},g^{2},\ldots, g^{d-1}, \widetilde{h},\widetilde{g}^{1},\widetilde{g}^{2},\ldots,\widetilde{g}^{d-1} $ *have the perfect reconstruction property*, *then the sub*-*band operator* *T* *is a bounded linear operator and reversible on* $l^{2}(-\infty,+\infty)$.

The proof of Theorem 2.1 is trivial.

As is well known, a bounded linear operator on $l^{2}(-\infty,+\infty)$ can be expressed by an infinite-dimensional matrix. Using matrix notations, we have a more helpful expression of the operator *T*. Let

$$s_{n,k}=\left [ \textstyle\begin{array}{c} h_{k-dn} \\ g^{1}_{k-dn} \\ g^{2}_{k-dn} \\ \vdots\\ g^{d-1}_{k-dn} \end{array}\displaystyle \right ],\qquad \widetilde{s}_{n,k}=\left [ \textstyle\begin{array}{c} \widetilde{h}_{k-dn} \\ \widetilde{g}^{1}_{k-dn} \\ \widetilde{g}^{2}_{k-dn} \\ \vdots\\ \widetilde{g}^{d-1}_{k-dn} \end{array}\displaystyle \right ]. $$

Then $A= [s_{n,k}]$ and $\widetilde{A}= [\widetilde{s}_{n,k}]$ ($-\infty< n,k<+\infty$) are infinite block circular matrices along four directions (up, down, left and right). At this time,

$$y=Ax, $$

where *x* and *y* are doubly infinite column vectors to fit the matrix operation. Hence *T* can be viewed as an infinite matrix *A*, i.e., $T=A$. If the sub-band decomposition has the perfect reconstruction condition, then it is obvious that *A* and *Ã* should satisfy

2.3 $$ A\widetilde{A}^{*}=\widetilde{A}^{*}A=I, $$

where *I* denotes the infinite identity matrix and $A^{*} $ denotes the transpose of complex conjugate of *A*. Thus, $T^{-1}=\widetilde{A}^{*}$.

Since $(Ax,y)=(x,A^{*}y)$, the adjoint operator of *T* is $T^{*}=A^{*}$. Let $Q=T^{*}T$, we have the following lemma.

### Lemma 2.1

*Retaining the definitions and notations as above*, *we have*

$$\begin{gathered} (1) \quad\|Q\|=\|T\|^{2}; \\ (2) \quad T^{-1}=\widetilde{T}^{*}=\widetilde{A}^{*},\qquad \widetilde {T}^{-1}=T^{*}=A^{*}; \\ (3)\quad\|Q\|=\sup_{\|x\|=1}\bigl\{ \big|(Qx,x) \big|\bigr\} . \end{gathered} $$

### Proof

Items (1) and (2) are trivial according to the operator theory [18]. Item (3) follows the fact that *Q* is a self-adjoint operator due to $Q^{*}=Q$. □

$Q=T^{*}T$ is called a frame operator in general [2]. Let $\nu_{n}$ and $\widetilde{\nu}_{n}$ denote the *n*th rows of *A* and *Ã*, respectively. Clearly, $(\nu_{j} ,\widetilde{\nu}_{k})=\delta_{j-k}$, where $\delta_{j}$ is the Dirac sequence, i.e., $\delta_{j}=1 $ for $j=0$ otherwise $\delta_{j}=0 $. Therefore, $\{ \nu_{n}\}$ and $\{ \widetilde{\nu}_{n}\}$ are dual biorthogonal bases in $l^{2}(-\infty,+\infty)$. Let $e_{n}\in l^{2}(-\infty,+\infty)$, its *n*th component be 1 and otherwise be 0. Then $T\widetilde{\nu}_{n}=e_{n}=\widetilde{T}\nu_{n}$. For an arbitrary $x\in l^{2}(-\infty,+\infty)$,

$$\begin{gathered} x=\sum_{n=-\infty}^{+\infty}(x,\nu_{n}) \widetilde{\nu}_{n}=\sum_{n=-\infty }^{+\infty}(x, \widetilde{\nu}_{n})\nu_{n}, \\ Qx=\sum_{n=-\infty}^{+\infty}(x,\nu_{n})Q \widetilde{\nu}_{n}=\sum_{n=-\infty }^{+\infty}(x, \nu_{n})T^{*}e_{n}=\sum_{n=-\infty}^{+\infty}(x, \nu _{n})T^{*}\widetilde{T}\nu_{n} =\sum _{n=-\infty}^{+\infty}(x,\nu_{n})\nu_{n}.\end{gathered} $$

Let *m* and *M* denote the lower bound and the upper bound of $\|T\|$, respectively.

$$\begin{gathered} Tx=\sum_{n=-\infty}^{+\infty}(x,\nu_{n})T \widetilde{\nu}_{n}=\sum_{n=-\infty }^{+\infty}(x, \nu_{n})e_{n}, \\ \|Tx\|^{2}=\sum_{n=-\infty}^{+\infty}\big|(x, \nu_{n})\big|^{2}.\end{gathered} $$

Then

2.4 $$ m^{2}\|x\|^{2}\leq\sum_{n=-\infty}^{+\infty}\big|(x, \nu _{n})\big|^{2}\leq M^{2}\|x\|^{2}. $$

Similarly,

2.5 $$ \widetilde{m}^{2}\|x\|^{2}\leq\sum _{n=-\infty}^{+\infty }\big|(x,\widetilde{\nu}_{n})\big|^{2} \leq\widetilde{M}^{2}\|x\|^{2} , $$

where *m̃* and *M̃* are the lower bound and the upper bound of $\|\widetilde{T}\|$, respectively. Therefore, (2.4) and (2.5) are the counterparts of (1.1) in $l^{2}(-\infty,+\infty)$.

Now we define a finite matrix $A_{n}$ as the partial matrix of the infinite matrix *A*, its row index and column index are finite with the following form:

$$A_{n}=\left [ \textstyle\begin{array}{c@{\quad}c@{\quad}c@{\quad}c@{\quad}c@{\quad}c@{\quad }c@{\quad}c@{\quad}c@{\quad}c} h_{0} & h_{1} & h_{2} &\ldots& h_{d-1} & \cdots& 0 & 0 & 0 & 0\\ g^{1}_{0} & g^{1}_{1} & g^{1}_{2} &\ldots& g^{1}_{d-1} & \cdots& 0 & 0 & 0 & 0\\ g^{2}_{0} & g^{2}_{1} & g^{2}_{2} & \ldots&g^{2}_{d-1} & \cdots& 0 & 0 & 0 & 0\\ g^{d-1}_{0} &g^{d-1}_{1} & g^{d-1}_{2} &\ldots& g^{d-1}_{d-1} & \cdots& 0 & 0 & 0 & 0\\ h_{-d} & \ldots& h_{-3} & h_{-2} & h_{-1}& \cdots&0 & 0 & 0 & 0\\ g^{1}_{-d} &\ldots& g^{1}_{-3} & g^{1}_{-2} & g^{1}_{-1}& \cdots& 0 & 0 & 0 & 0 \\ g^{2}_{-d} & \ldots&g^{2}_{-3} & g^{2}_{-2} & g^{2}_{-1}& \cdots& 0 & 0 & 0 & 0 \\ g^{d-1}_{-d}& \ldots& g^{d-1}_{-3} & g^{d-1}_{-2} & g^{d-1}_{-1}& \cdots& 0 & 0 & 0 & 0 \\ \cdots&\cdots& \cdots& \cdots& \cdots& \cdots& \cdots& \cdots& \cdots&\cdots\\ 0 & 0 & 0 & 0 & \cdots& h_{0} & h_{1} & h_{2} & \ldots&h_{d-1} \\ 0 & 0 & 0 & 0 & \cdots&g^{1}_{0} & g^{1}_{1} & g^{1}_{2} &\ldots& g^{1}_{d-1}\\ 0 & 0 & 0 & 0 & \cdots& g^{2}_{0} & g^{2}_{1} & g^{2}_{2} &\ldots& g^{2}_{d-1}\\ 0 & 0 & 0 & 0 & \cdots& g^{d-1}_{0} & g^{d-1}_{1} & g^{d-1}_{2} &\ldots& g^{d-1}_{d-1}\\ 0 & 0 & 0 & 0& \cdots& h_{-d} &\ldots& h_{-3} & h_{-2} & h_{-1} \\ 0 & 0 & 0 & 0 & \cdots&g^{1}_{-d}&\ldots& g^{1}_{-3} & g^{1}_{-2} & g^{1}_{-1}\\ 0 & 0 & 0 & 0 & \cdots&g^{2}_{-d} &\ldots& g^{2}_{-3} & g^{2}_{-2} & g^{2}_{-1}\\ 0 & 0 & 0 & 0 & \cdots&g^{d-1}_{-d}&\ldots& g^{d-1}_{-3} & g^{d-1}_{-2} & g^{d-1}_{-1} \end{array}\displaystyle \right ]. $$

### Theorem 2.2

*Let* $Q_{n}=A_{n}^{*}A_{n}$ *and* $\{\tau_{1}^{(n)},\tau_{2}^{(n)},\ldots, \tau_{dn}^{(n)}\}$ *be all eigenvalues of* $Q_{n}$. *Then*

$$\|Q\|=\lim_{n\rightarrow\infty}\max\bigl\{ \tau_{1}^{(n)}, \tau_{2}^{(n)},\ldots, \tau_{dn}^{(n)}\bigr\} . $$

### Proof

Since $Q_{n}$ is a positive operator, it has *dn* positive real eigenvalues, and $(Qx,x)\geq0$. From Lemma 2.1, we have

$$\|Q\|=\sup_{\|x\|=1}\bigl\{ \big|(Qx,x)\big|\bigr\} =\sup _{\|x\|=1}\bigl\{ (Qx,x)\bigr\} . $$

Hence, there exists an $x^{(m)}\in l^{2}(-\infty,+\infty)$, such that $\|x^{(m)}\|=1$ and $\|Q\|= \lim_{m\rightarrow\infty}(Qx^{(m)},x^{(m)})$. Let *dn*-dimensional vector $[x]_{n}^{(m)}$ be the finite part of $x^{(m)}$.

On one hand, clearly, $\| [x]_{n}^{(m)}\| \leq\|x^{(m)}\|$ for an arbitrary *n*. Let $[y]_{n}$ be a *dn*-dimensional vector. Then

$$ \|Q\|=\lim_{m\rightarrow\infty}\bigl(Qx^{(m)},x^{(m)}\bigr)= \lim_{m\rightarrow\infty}\lim_{n\rightarrow\infty}\bigl(Q_{n} [x]_{n}^{(m)}, [x]_{n}^{(m)} \bigr). $$

Since $Q_{n}$ is a finite-dimensional self-adjoint compact operator, we have

$$\sup_{\| [y]_{n}\|\leq 1}\bigl\{ \bigl(Q_{n} [y]_{n}, [y]_{n} \bigr)\bigr\} =\max\bigl\{ \tau_{1}^{(n)},\tau_{2}^{(n)}, \ldots, \tau_{dn}^{(n)}\bigr\} . $$

Hence,

$$\|Q\|\leq\lim_{n\rightarrow \infty}\max\bigl\{ \tau_{1}^{(n)}, \tau_{2}^{(n)},\ldots, \tau_{dn}^{(n)}\bigr\} . $$

On the other hand, for every sufficiently large *n*, there exists a *dn*-dimensional vector $[y]_{n}$, such that $\| [y]_{n}\|=1$, and

$$\sup_{\|y\|=1}\bigl\{ \bigl(Q_{n} [y], [y]\bigr)\bigr\} = \bigl(Q_{n} [y]_{n}, [y]_{n}\bigr)=\max\bigl\{ \tau_{1}^{(n)},\tau _{2}^{(n)},\ldots, \tau_{dn}^{(n)}\bigr\} . $$

Extend $[y]_{n}$ to $y^{(n)}\in l^{2}(-\infty,+\infty)$ by appending 0s to the tuples which are not defined by $[y]_{n}$. Clearly, $\| [y]_{n}\|=\|y^{(n)}\|=1$, and

$$\|Q\|=\sup_{\|x\|=1}\bigl\{ (Qx,x)\bigr\} \geq \bigl(Qy^{(n)},y^{(n)} \bigr)=\bigl(Q [y]_{n}, [y]_{n}\bigr)=\max\bigl\{ \tau_{1}^{(n)},\tau_{2}^{(n)},\ldots, \tau_{dn}^{(n)}\bigr\} . $$

It implies that

$$\|Q\|\geq\lim_{n\rightarrow \infty}\max\bigl\{ \tau_{1}^{(n)}, \tau_{2}^{(n)},\ldots, \tau_{dn}^{(n)}\bigr\} . $$

Hence, $\|Q\|= \lim_{n\rightarrow \infty}\max\{\tau_{1}^{(n)},\tau_{2}^{(n)},\ldots, \tau_{dn}^{(n)}\}$. The proof is complete. □

Theoretically, Theorem 2.2 gives an exact value for $\|Q\|$ and $\|T\|=\sqrt{\|Q\|}$ is used to compute the norm of *T*. However, the eigenvalues are not easy to compute for generalized block Toeplitz matrices. We shall use the theory of circular matrix to compute the norm of *Q*. The block circular matrix is defined as follows [19]:

$$B_{n}=\left [ \textstyle\begin{array}{c@{\quad}c@{\quad}c@{\quad}c@{\quad}c@{\quad}c@{\quad }c@{\quad}c@{\quad}c@{\quad}c@{\quad}c} h_{0} & h_{1} & h_{2} &\ldots& h_{d-1} & \cdots& h_{-d} &\ldots& h_{-3} & h_{-2} & h_{-1} \\ g^{1}_{0} & g^{1}_{1} & g^{1}_{2} &\ldots& g^{1}_{d-1} & \cdots& g^{1}_{-d} &\ldots& g^{1}_{-3} & g^{1}_{-2} & g^{1}_{-1} \\ g^{2}_{0} & g^{2}_{1} & g^{2}_{2} &\ldots& g^{2}_{d-1}& \cdots& g^{2}_{-d}&\ldots& g^{2}_{-3} &g^{2}_{-2} & g^{2}_{-1}\\ g^{d-1}_{0} & g^{d-1}_{1} &\ldots& g^{d-1}_{2} & g^{d-1}_{d-1} & \cdots& g^{d-1}_{-d} &\ldots& g^{d-1}_{-3} & g^{d-1}_{-2} &g^{d-1}_{-1}\\ h_{-d}&\ldots& h_{-3} &h_{-2} & h_{-1}& \cdots&h_{-2d}&\ldots& h_{-7} & h_{-6} & h_{-5}\\ g^{1}_{-d}&\ldots& g^{1}_{-3} & g^{1}_{-2} & g^{1}_{-1} &\cdots& g^{1}_{-2d}&\ldots& g^{1}_{-7} & g^{1}_{-6} & g^{1}_{-5}\\ g^{2}_{-d}&\ldots& g^{2}_{-3} & g^{2}_{-2} & g^{2}_{-1} &\cdots& g^{2}_{-2d}&\ldots& g^{2}_{-7} & g^{2}_{-6} & g^{2}_{-5}\\ g^{d-1}_{-d}&\ldots& g^{d-1}_{-3} & g^{d-1}_{-2} & g^{d-1}_{-1} &\cdots& g^{d-1}_{-2d}&\ldots& g^{d-1}_{-7} & g^{d-1}_{-6} & g^{d-1}_{-5}\\ \cdots&\cdots& \cdots& \cdots& \cdots& \cdots& \cdots& \cdots& \cdots& \cdots& \cdots\\ h_{2d}& h_{2d+1} & h_{2d+2} &\ldots& h_{3d-1}& \cdots&h_{d} & h_{d+1} & h_{d+2} &\ldots& h_{2d-1} \\ g^{1}_{2d} & g^{1}_{2d+1} &g^{1}_{2d+2} &\ldots& g^{1}_{3d-1}& \cdots& g^{1}_{d} & g^{1}_{d+1} &g^{1}_{d+2} &\ldots& g^{1}_{2d-1}\\ g^{2}_{2d} & g^{2}_{2d+1} &g^{2}_{2d+2} &\ldots& g^{2}_{3d-1} & \cdots& g^{2}_{d} & g^{2}_{d+1} &g^{2}_{d+2} &\ldots& g^{2}_{2d-1} \\ g^{d-1}_{2d} & g^{d-1}_{2d+1} &g^{d-1}_{2d+2} &\ldots& g^{d-1}_{3d-1} & \cdots& g^{d-1}_{d} & g^{d-1}_{d+1} &g^{d-1}_{d+2} &\ldots& g^{d-1}_{2d-1}\\ h_{d} & h_{d+1} & h_{d+2} &\ldots& h_{2d-1} & \cdots& h_{0} & h_{1} & h_{2} &\ldots& h_{d-1}\\ g^{1}_{d} & g^{1}_{d+1} &g^{1}_{d+2} &\ldots& g^{1}_{2d-1} & \cdots& g^{1}_{0} & g^{1}_{1} & g^{1}_{2} &\ldots& g^{1}_{d-1}\\ g^{2}_{d} & g^{2}_{d+1} &g^{2}_{d+2} &\ldots& g^{2}_{2d-1} & \cdots& g^{2}_{0} & g^{2}_{1} & g^{2}_{2} &\ldots& g^{2}_{d-1}\\ g^{d-1}_{d} & g^{d-1}_{d+1} &g^{d-1}_{d+2} &\ldots& g^{d-1}_{2d-1} & \cdots& g^{d-1}_{0} & g^{d-1}_{1} &g^{d-1}_{2} &\ldots& g^{d-1}_{d-1} \end{array}\displaystyle \right ]. $$

Clearly, $A_{n}$ is different from $B_{n}$. Let $C_{n}=B_{n}-A_{n}$, then only finitely many (fixed) elements in $C_{n}$ are not 0 no matter how large the dimension 4*n* is. We have

2.6 $$ A_{n}^{*}A_{n}=B_{n}^{*}B_{n}-C_{n}^{*}B_{n}-B_{n}^{*}C_{n}+C_{n}^{*}C_{n}. $$

We have Theorem 2.3.

### Theorem 2.3

*Let* $P_{n}=B_{n}^{*}B_{n}$ *and* $\{\lambda_{1}^{(n)},\lambda_{2}^{(n)},\ldots, \lambda_{dn}^{(n)}\}$ *be all eigenvalues of* $P_{n}$. *Then*

$$\|Q\|=\lim_{n\rightarrow\infty}\max\bigl\{ \lambda_{1}^{(n)}, \lambda _{2}^{(n)},\ldots, \lambda_{dn}^{(n)} \bigr\} . $$

### Proof

Let $[x]_{n}$ be a *dn*-dimensional vector. We first prove that for $\| [x]_{n}\|=1$, $\lim_{n\rightarrow \infty}|(C_{n}^{*}B_{n} [x]_{n}, [x]_{n})|=0$, $\lim_{n\rightarrow\infty}|(B_{n}^{*}C_{n} [x]_{n}, [x]_{n})|=0$, $\lim_{n\rightarrow\infty}|(C_{n}^{*}C_{n} [x]_{n}, [x]_{n})|=0$.

In fact, the operator $B_{n}$ is bounded. For $\| [x]_{n}\|=1$, there exists a positive real number *M* such that

$$ \begin{aligned}\big|\bigl(C_{n}^{*}B_{n} [x]_{n}, [x]_{n} \bigr)\big|&=\big|\bigl(B_{n} [x]_{n},C_{n} [x]_{n} \bigr)\big| \\ &\leq \big\| B_{n} [x]_{n}\big\| \big\| C_{n} [x]_{n} \big\| \leq M\big\| C_{n} [x]_{n}\big\| . \end{aligned} $$

Note that $C_{n} [x]_{n}$ only contains *dn* nonzero components.

Hence $\lim_{n\rightarrow \infty}\|C_{n} [x]_{n}\|=0$, i.e., $\lim_{n\rightarrow \infty}|(C_{n}^{*}C_{n} [x]_{n}, [x]_{n})|=0$.

Similarly, we can verify $\lim_{n\rightarrow \infty}|(B_{n}^{*}C_{n} [x]_{n}, [x]_{n})|=0$ and $\lim_{n\rightarrow\infty}|(C_{n}^{*}C_{n} [x]_{n}, [x]_{n})|=0$.

It follows from (2.6) that

$$\bigl(Q_{n} [x]_{n}, [x]_{n}\bigr)= \bigl(P_{n} [x]_{n}, [x]_{n}\bigr)+ \bigl(R_{n} [x]_{n}, [x]_{n}\bigr), $$

where $R_{n}=-C_{n}^{*}B_{n}-B_{n}^{*}C_{n}+C_{n}^{*}C_{n}$ and $\lim_{n\rightarrow\infty}|(R_{n}, [x]_{n})|=0$.

It implies that

$$\lim_{n\rightarrow \infty}\sup_{\| [x]_{n}\|=1}\bigl\{ \bigl(Q_{n} [x]_{n}, [x]_{n}\bigr)\bigr\} =\lim _{n\rightarrow \infty}\sup_{\| [x]_{n}\|=1}\bigl\{ \bigl(P_{n} [x]_{n}, [x]_{n} \bigr)\bigr\} . $$

By Theorem 2.2, we have

$$\|Q\|=\lim_{n\rightarrow\infty}\max\bigl\{ \tau_{1}^{(n)}, \tau_{2}^{(n)},\ldots, \tau_{dn}^{(n)}\bigr\} = \lim_{n\rightarrow \infty}\max\bigl\{ \lambda_{1}^{(n)}, \lambda_{2}^{(n)},\ldots, \lambda_{dn}^{(n)} \bigr\} . $$

The proof is complete. □

A so-called *d*-circular matrix [20], which is generated by the filters $h,g^{1},g^{2},\ldots,g^{d-1}$, is denoted as $M_{n} $. For $d=4$, $M_{3} $ is as follows:

$$M_{3}=\left [ \textstyle\begin{array}{c@{\quad}c@{\quad}c@{\quad}c@{\quad}c@{\quad}c@{\quad }c@{\quad}c@{\quad}c@{\quad}c@{\quad}c@{\quad}c} h_{0} & h_{1} & h_{2} & h_{3} & 0 & 0 & 0 &0 &h_{-4} & h_{-3} & h_{-2} & h_{-1}\\ h_{-4} & h_{-3} &h_{-2} & h_{-1} & h_{0} & h_{1}&h_{2} & h_{3}& 0 & 0& 0 & 0\\ 0 & 0 & 0 & 0 & h_{-4} & h_{-3} &h_{-2} & h_{-1} & h_{0} & h_{1} & h_{2} & h_{3} \\ g^{1}_{0} & g^{1}_{1} & g^{1}_{2} & g^{1}_{3} & 0 & 0 & 0 & 0& g^{1}_{-4} & g^{1}_{-3} & g^{1}_{-2} & g^{1}_{-1}\\ g^{1}_{-4} & g^{1}_{-3} & g^{1}_{-2} & g^{1}_{-1}& g^{1}_{0} & g^{1}_{1} & g^{1}_{2} & g^{1}_{3}& 0 & 0 & 0 & 0\\ 0 & 0 & 0 & 0 & g^{1}_{-4} & g^{1}_{-3} & g^{1}_{-2} & g^{1}_{-1}& g^{1}_{0} & g^{1}_{1} & g^{1}_{2} & g^{1}_{3}\\ g^{2}_{0} & g^{2}_{1} & g^{2}_{2} & g^{2}_{3} & 0 & 0 & 0 & 0& g^{2}_{-4} & g^{2}_{-3} &g^{2}_{-2} & g^{2}_{-1}\\ g^{2}_{-4} & g^{2}_{-3} & g^{2}_{-2} & g^{2}_{-1}& g^{2}_{0} & g^{2}_{1} & g^{2}_{2} & g^{2}_{3} & 0 & 0 & 0 & 0\\ 0 & 0 & 0 & 0 & g^{2}_{-4} & g^{2}_{-3} & g^{2}_{-2} & g^{2}_{-1}& g^{2}_{0} & g^{2}_{1} & g^{2}_{2} & g^{2}_{3}\\ g^{3}_{0} & g^{3}_{1} & g^{3}_{2} & g^{3}_{3} & 0 & 0 & 0 & 0&g^{3}_{-4}& g^{3}_{-3} & g^{3}_{-2} &g^{3}_{-1}\\ g^{3}_{-4} & g^{3}_{-3} & g^{3}_{-2} & g^{3}_{-1}& g^{3}_{0} & g^{3}_{1} & g^{3}_{2} & g^{3}_{3} & 0 & 0 & 0 & 0\\ 0 & 0 & 0 & 0& g^{3}_{-4} & g^{3}_{-3} & g^{3}_{-2} & g^{3}_{-1}& g^{3}_{0} & g^{3}_{1} & g^{3}_{2} & g^{3}_{3} \end{array}\displaystyle \right ]. $$

Clearly, $B_{n} $ and $M_{n} $ are not so different. One can be obtained by exchanging the places of some rows of another, i.e., there exists an orthonormal matrix $E_{n} $ such that $M_{n}=E_{n}B_{n} $. The purpose is to facilitate the calculation of the eigenvalues.

Similarly, let $\widetilde{M}_{n} $ be a *d*-circular matrix generated by $\widetilde{h},\widetilde{g}^{1},\widetilde{g}^{2},\ldots,\widetilde{g}^{d-1}$. The perfect reconstruction condition is that there exists an integer $N_{0}$, such that, for all $n\geq N_{0}$ (in what follows, *n* sufficiently large is in this sense),

2.7 $$ M_{n}{\widetilde{M}}_{n}^{*}=I_{dn}, $$

where $I_{dn}$ is a $dn\times dn$ identity matrix.

### Theorem 2.4

*Let* $M_{n} $ *be a* *d*-*circular matrix generated by* $h,g^{1},g^{2},\ldots,g^{d-1}$. *For sufficiently large* *n*, *then*

2.8 $$ \max\{\lambda_{1},\lambda_{2},\ldots, \lambda_{dn} \} \leq \max\{C_{0},C_{1},\ldots,C_{d-1}\}, $$

*where*

$$\begin{gathered} C_{0}=\sum_{j}\bigg| \sum_{i}{h_{i}h_{i+dj}}\bigg|+\sum _{n=1}^{d-1}\sum_{j}\bigg| \sum_{i}{h_{i}g^{n}_{i+dj}}\bigg|, \\ C_{k}=\sum_{j}\bigg|\sum _{i}{g^{k}_{i}h_{i+dj}}\bigg|+\sum _{n=1}^{d-1}\sum_{j}\bigg| \sum_{i}{g^{k}_{i}g^{n}_{i+dj}}\bigg|,\quad 1\leq k\leq d-1. \end{gathered} $$

### Proof

Define

$$\|A_{dn}\|_{\infty}=\max_{1\leq i\leq dn}\sum ^{dn}_{j=1}|a_{i,j}|. $$

Since $\|A_{dn}B_{dn}\|_{\infty}\leq\|A_{dn} \|_{\infty}\times\|B_{dn}\|_{\infty}$, $\| \cdot \|_{\infty}$ is a compatible matrix norm. Note that $A_{dn}=M_{n}M_{n}^{T}$ positive definite matrices, and all of the eigenvalues of $M_{n}M_{n}^{T}$ are positive. According to the theory of matrices [19], we have

$$|\lambda_{i}|\leq\|A_{dn}\|_{\infty}=\max _{1\leq i\leq dn}\sum^{dn}_{j=1}|a_{i,j}|,\quad i=1,2,\ldots,dn. $$

Note that the sub-matrices of $M_{n}M_{n}^{T}$ such as $HH^{T},HG_{1}^{T},HG_{2}^{T},\ldots,HG_{d-1}^{T},\ldots $ are all 1-circular matrices. Then

$$\sum^{kn+n}_{j=kn+1}|a_{kn+1,j}|=\sum ^{kn+n}_{j=kn+1}|a_{kn+2,j}| =\cdots=\sum ^{kn+n}_{j=kn+1}|a_{kn+n,j}|, \quad 0\leq k\leq d-1. $$

We have

$$C_{0}=\sum^{dn}_{j=1}|a_{1,j}|= \sum_{j}\bigg|\sum_{i}{h_{i}h_{i+dj}}\bigg|+ \sum_{n=1}^{d-1}\sum _{j}\bigg|\sum_{i}{h_{i}g^{n}_{i+dj}}\bigg|. $$

Firstly, we verify that, for $n\geq N_{0}$,

$$\sum^{n}_{j=1}|a_{1,j}|\leq\sum _{j}\bigg|\sum_{i}{h_{i}h_{i+dj}}\bigg|. $$

1. There exists $N^{*}_{0}$: when $n\geq N^{*}_{0}\geq N_{0}$, we have
  $$HH^{T}= \Bigl[\sum h_{i}h_{i},\sum h_{i}h_{i+d},\sum h_{i}h_{i+2d}, \ldots,0,\ldots ,\sum h_{i}h_{i+d} \Bigr]. $$
  Note that when the dimension increases, the nonzero elements of $HH^{T}$ are the same. Therefore
  $$\sum^{n}_{j=1}|a_{1,j}| = \sum _{j}\bigg|\sum_{i}{h_{i}h_{i+dj}}\bigg|. $$
2. When $N_{0}\leq n\leq N^{*}_{0} $, the nonzero elements of $HH^{T}$ decrease.
  It follows from $|a+b|\leq|a|+|b|$ that
  $$\sum^{n}_{j=1}|a_{1,j}|\leq\sum _{j}\bigg|\sum_{i}{h_{i}h_{i+dj}}\bigg|. $$
  We obtain
  $$\sum^{n}_{j=1}|a_{1,j}|\leq\sum _{j}\bigg|\sum_{i}{h_{i}h_{i+dj}}\bigg|. $$
  Similarly, (2.8) holds true.

 □

### Remark

The right-hand side of (2.8) is only determined by the filters.

### Theorem 2.5

*Let* *T* *be the sub*-*band operator of* *d*-*band wavelets*. *Then*

2.9 $$\begin{aligned}& \frac{1}{\sqrt{\max\{\widetilde{C}_{0},\widetilde{C}_{1},\ldots ,\widetilde{C}_{d-1}\}}}\leq\|T\|\leq \sqrt{\max\{C_{0},C_{1}, \ldots,C_{d-1}\}}, \end{aligned}$$

2.10 $$\begin{aligned}& \frac{1}{\sqrt{\max\{C_{0},C_{1},\ldots,C_{d-1}\}}}\leq\big\| T^{-1}\big\| \leq \sqrt{\max\{\widetilde{C}_{0}, \widetilde{C}_{1},\ldots,\widetilde {C}_{d-1}\}}, \end{aligned}$$

*where the filter bands are* $\{h,g^{1},g^{2},\ldots,g^{d-1}\} $ *and* $\{\widetilde{h},\widetilde{g}^{1},\widetilde{g}^{2},\ldots,\widetilde {g}^{d-1}\}$, *respectively*,

$$\begin{aligned}& C_{0}=\sum_{j}\bigg| \sum_{i}{h_{i}h_{i+dj}}\bigg|+\sum _{n=1}^{d-1}\sum_{j}\bigg| \sum_{i}{h_{i}g^{n}_{i+dj}}\bigg|, \\ & C_{k}=\sum_{j}\bigg|\sum _{i}{g^{k}_{i}h_{i+dj}}\bigg|+\sum _{n=1}^{d-1}\sum_{j}\bigg| \sum_{i}{g^{k}_{i}g^{n}_{i+dj}}\bigg|,\quad 1 \leq k\leq d-1, \\& \widetilde{C}_{0}=\sum_{j}\bigg|\sum _{i}{\widetilde{h}_{i}\widetilde {h}_{i+dj}}\bigg|+\sum_{n=1}^{d-1}\sum _{j}\bigg|\sum_{i}{ \widetilde{h}_{i}\widetilde{g}^{n}_{i+dj}}\bigg|, \\ & \widetilde{C}_{k}=\sum_{j}\bigg|\sum _{i}{\widetilde{g}^{k}_{i} \widetilde {h}_{i+dj}}\bigg|+\sum_{n=1}^{d-1} \sum_{j}\bigg|\sum_{i}{ \widetilde{g}^{k}_{i}\widetilde{g}^{n}_{i+dj}}\bigg|,\quad 1 \leq k\leq d-1. \end{aligned}$$

### Proof

By Theorem 2.3, we have

$$\|T\|^{2}=\|Q\|=\lim_{n\rightarrow\infty}\max\{ \lambda_{1},\lambda_{2},\ldots , \lambda_{dn}\}, $$

where $\{\lambda_{1},\lambda_{2},\ldots, \lambda_{dn}\}$ are all eigenvalues of $M_{n}M^{T}_{n}$.

According to Theorem 2.4, we have

$$\|T\|=\sqrt{\|Q\|}\leq \sqrt{\max\{C_{0},C_{1}, \ldots,C_{d-1}\}}. $$

Similarly,

$$\big\| T^{-1}\big\| \leq\sqrt{\max\{\widetilde{C}_{0}, \widetilde{C}_{1},\ldots ,\widetilde{C}_{d-1}\}}. $$

The proof is complete. □

## Examples

In this section, we present two examples to illustrate the proposed results.

### Example 3.1

Let the lengths of scaling filters be $(15,9)$. Assume that the scaling symbols $(H_{0}(z),\widetilde{H}_{0}(z)) $ have the following form:

$$\begin{gathered} H_{0}(z)= \biggl(\frac{1+z+z^{2}}{3} \biggr)^{5}Q(z), \\ \widetilde{H}_{0}(z)= \biggl(\frac{1+z+z^{2}}{3} \biggr)^{3} \widetilde{Q}(z), \end{gathered} $$

where $(Q(z),\widetilde{Q}(z))$ are symmetric Laurent polynomials with degree $(4,2)$.

**Figure 1:**
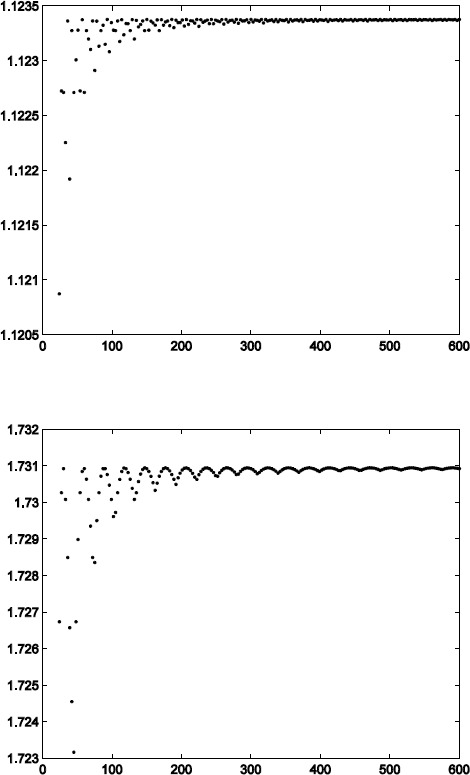
The spectral radius of $MM^{T}$ and $\widetilde{M}\widetilde {M}^{T}$, respectively (from top to bottom)

We can obtain the associated scaling filters as follows [21]:

$$\begin{gathered} h_{0}\approx [ 0.0302708750, 0.0197271260, 0.0109853080, -0.12261759, 0.011382944,\\ \phantom{h_{0}\approx\ } 0.24928687, 0.83207454, 0.93777986 ], \\ \widetilde{h}_{0}\approx [ -0.20140256, -0.090291448, 0.13193077, 1.0694718, 1.1805829 ] \end{gathered} $$

(whereas the other half is symmetric and so is skipped). Thus, the wavelet filters $h_{1}$, $h_{2}$ and $\widetilde{h}_{1}$, $\widetilde{h}_{2}$ can be obtained as follows:

$$\begin{aligned}& h_{1}\approx [ 0.032348717, 0.021081228, 0.011739358, -0.75426796,1.3781973], \\ & h_{2}\approx4 [ -0.044296947, -0.028867731, -0.016075373, 0.29594173, 0], \\& \widetilde{h}_{1}\approx [-0.0047182543, -0.0021152562, 0.0030907400,0.038680809,0.033766352,\\& \phantom{\widetilde{h}_{1}\approx\ } 0.016128444, -0.75722871, 1.3447918], \\& \widetilde{h}_{2}\approx [ 0.14107656, 0.063246501,-0.092413623,-0.45441064, -0.69483538,\\& \phantom{\widetilde{h}_{2}\approx\ } {-}0.94219467, 4.8815912,0]/4 \end{aligned}$$

(whereas the other half is symmetric/antisymmetric and so skipped). See Fig. 1 for the spectral radius of $M_{n}M_{n}^{T}$ and $\widetilde{M}_{n}\widetilde{M}_{n}^{T}$. From Theorem 2.3, we can obtain $\|T\|\approx1.06$, which is an approximation and not an exact value. Similarly, $\|T^{-1}\|\approx1.32$. By Theorem 2.5, we can calculate that $0.66\leq\|T\|\leq1.15$ and $0.94\leq\|T^{-1}\|\leq1.51$.

### Example 3.2

In [20], a 4-band symmetric biorthogonal wavelets which is denoted as Op(12-12) is designed. The corresponding wavelet filter banks $\{h,g^{1}, g^{2}, g^{3} \}$ are as follows:

$$\left [ \textstyle\begin{array}{c@{\quad}c@{\quad}c@{\quad}c@{\quad}c@{\quad}c@{\quad }c@{\quad}c@{\quad}c@{\quad}c@{\quad}c@{\quad}c} t_{0} & t_{1} & t_{2} & t_{3} & t_{4} & t_{5} & t_{5} & t_{4} & t_{3} & t_{2} & t_{1} & t_{0} \\ t_{1} & -t_{0} & -t_{3} & t_{2} & t_{5} & -t_{4} & -t_{4} & t_{5} & t_{2} & -t_{3} & -t_{0} & t_{1} \\ \widetilde{t}_{0} & -\widetilde{t}_{1} &\widetilde{t}_{2} & -\widetilde{t}_{3} & \widetilde{t}_{4}& -\widetilde{t}_{5} & \widetilde{t}_{5} & -\widetilde{t}_{4} & \widetilde{t}_{3} & -\widetilde{t}_{2} &\widetilde{t}_{1} & -\widetilde {t}_{0} \\ \widetilde{t}_{1} & \widetilde{t}_{0} & -\widetilde{t}_{3} & -\widetilde {t}_{2} & \widetilde{t}_{5}& \widetilde{t}_{4} & -\widetilde{t}_{4} & -\widetilde{t}_{5} & \widetilde{t}_{2} & \widetilde{t}_{3} & -\widetilde{t}_{0} & -\widetilde{t}_{1} \end{array}\displaystyle \right ], $$

where $t_{0}=0.01129264$, $t_{1}=-0.01660958$, $t_{2}= -0.01418315$, $t_{3}= 0.02102888 $, $t_{4}= 0.4676785$, $t_{5}=0.5307927$, $\widetilde{t}_{0}= -0.07653$, $\widetilde{t}_{1}=-0.04528$, $\widetilde{t}_{2}=0.01722 $, $\widetilde{t}_{3}= 0.11097$, $\widetilde{t}_{4}= 0.46556$, $\widetilde{t}_{5}= 0.52806$.

**Figure 2:**
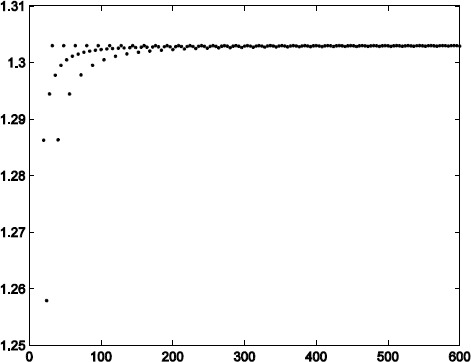
The spectral radius of $MM^{T}$ ($\widetilde{M}\widetilde{M}^{T}$)

It has been shown that the eigenvalues of $M^{T}_{4n} M_{4n}$ appear in pairs of reciprocal, $M^{T}_{4n} M_{4n}$ and $\widetilde{M}^{T}_{4n} \widetilde{M}_{4n}$ have the same eigenvalues [20]. It is obvious that $\max\{\lambda_{i}\}=\max\{\widetilde{\lambda}_{i}\}=\frac{1}{\min\{\lambda _{i}\}}=\frac{1}{\min\{\widetilde{\lambda}_{i}\}} $. See Fig. 2 for the spectral radius of $M_{n}M_{n}^{T}$ and $\widetilde{M}_{n}\widetilde{M}_{n}^{T}$. From Theorem 2.3, we can obtain $\|T\|\approx1.14$, which is an approximation and not an exact value. According to Theorem 2.5, we have $\|T\|=\|T^{-1}\|\leq1.18$.

## Conclusions and future work

We can obtain the upper bound and the lower bound or an approximation of the sub-band operator’s norm based on the theory of circular matrix which plays an important role. We will calculate their norm for some special symmetric wavelets and design a family of biorthogonal wavelets based on the size of the norm.
